# Flexural Performance of Direct Resin Composite Restorative Materials Past Expiration Date

**DOI:** 10.1055/s-0040-1709922

**Published:** 2020-05-12

**Authors:** Hiroko Nagaoka, Susan Bishop, Howard Roberts

**Affiliations:** 1 Division of Restorative Dentistry, University of Kentucky College of Dentistry, Lexington, Kentucky, United States; 2 Uniformed Services University of the Health Sciences, Bethesda, Maryland, United States

**Keywords:** resin restoration, shelf life, expiration date

## Abstract

**Objective** This study' s purpose was to examine the flexural properties of five direct restorative resin composites stored up to 30 months after the expiration date.

**Materials and Methods** Ambient-stored materials had pre-expiration date baseline flexure strength values as per ISO 4049 ( *n* = 20). All materials were used per manufacturer guidelines, photopolymerized on both sides using a LED-based visible light curing unit, and stored in 0.2M phosphate buffered saline. At 24 hours, specimens were stressed to failure in three-point bend at a 0.5 mm/min cross head speed. Additional samples were made at 3, 6, 9, 12, 15, 18, 24, and 30 months past expiration date. Young’s modulus (flexural) was ascertained using the linear slope of the stress-stain curve.

**Statistical Analysis** The mean data was found to contain a non-normal distribution and irregular variance which was compared using Kruskal–Wallis with Dunn’s posthoc testing.

Also, Pearson’s correlation analysis was used to identify possible similar degradation behavior between products within both flexure strength and modulus determinations. A 95% level of confidence (α = 0.05) was used.

**Results** Materials maintained similar to baseline flexure strength and modulus for up to 15 months past expiration date with two materials being similar at 30 months. However, clinicians were still advised to follow expiration dates, as resin composite degradation mechanisms are complex and vital constituents might degrade that are not overtly identified by clinical handling characteristics. No dental shelf life standards exist and manufacturers are requested to provide protocol information used in determining shelf life expiration.

## Introduction

Dental manufacturers publish a material expiration date intended to assure clinicians that purchased materials will maintain efficacy over a stated time period. 1 Manufacturers may use methods to help establish product shelf life, which is identified as the period between product formulation/manufacture and the time that the material no longer has the mechanical and physical properties required to accomplish its intended purpose. 1 Stability, somewhat synonymous with shelf life, is also described by the amount of the same characteristics which a product retains at the time of manufacture and throughout its storage period. 2 Various criteria were proposed to delineate stability assessment methods. For instance, pharmaceutical industry methods exemplify testing of biologic, mechanical, and optical properties. 3 However, other testing factors have been suggested that include the consideration of nonideal environmental conditions prior to and during product delivery. 4 Unfortunately, there is no established standard for the determination of dental product stability and shelf life.

For dental restorative products, stability and shelf-life determinations are frequently accomplished using accelerated aging protocols that expose materials to increased and/or more frequent heat and humidity conditions than the normally recommended ones. 4 5 6 Accelerated aging protocols usually follow the Arrhenius collision theory model that is known as the “10-degree rule,” 1 which presumes that a product’s reaction rate during storage will double for each 10°C increase above standard room temperature. 1 The Arrhenius model is based on the formula of r = Q10 (RT-ET/10), where r = accelerated aging rate, RT = room temperature (22°C), ET = elevated temperature (usually 37°C), and Q10 = reaction rate coefficient. Under this model 12 months of simulated ambient storage at 22°C would require 17 weeks storage at 37°C. However, hastened evaluations may not always be appropriate, as full-time, ambient storage shelf-life studies may be used for new materials not previously evaluated. 4 5 6

Resin composite shelf life determinations are complicated, as these materials contain many components in which individual constituent degradation could cause a potential myriad impact on the polymeric composite’s functional properties. 1 Furthermore, this individual degradation may be obscured by other structural components, until sufficient degradation accumulates, that affect material property. 3 7 Likewise, clinicians attempting to determine product shelf-life by assessing clinical handling characteristics alone is also not advised, as unobserved individual component degradation may not be discerned but could have an intense impact on the material’s functional longevity. 8 Hence, clinicians are advised to discard expired products. However, when based solely on arbitrary assigned criteria, this may represent additional costs to the dental profession. Due to a lack of standards, manufacturers are not necessarily required to divulge shelf-life determination methods. Therefore, some clinicians may be tempted and choose to use a product past its expiration date. 9

Studies involving expired resin composite performance reported various methodologies including flexure strength, 4 10 flexural modulus, 4 10 hardness, 9 11 diametral tensile strength, 12 surface roughness, 101 filler distribution, 13 thermal analysis, 10 infrared spectroscopy, 11 electron paramagnetic resonance (EPR) spectroscopy, 14 and X-ray diffraction. 14 Furthermore, accelerated and/or actual storage times differ, varying from 6 months ambient aging, 9 11 9 months simulated due to accelerated aging, 10 13 14 15 months, 12 and 7 years of ambient storage. 4

The purpose of this study was to investigate the postexpiration flexural strength and modulus of five, ambiently stored, visible light-cured direct restorative resin composites. The null hypothesis was that there would be no difference in the individual material’s flexure strength and flexural modulus as compared with that obtained 1 month prior to the expiration date.

## Materials and Methods

The products evaluated are listed in Table 1 . The materials used were in excess after other material testing and near manufacturer recommended expiration date. All five materials were stored in a laboratory storage drawer freely exposed to ambient conditions (23 ± 2°C, 52 ± 6% relative humidity) that were within manufacturer specified storage ranges. Baseline data was obtained with sample fabrication and testing 1 month prior to manufacturer’s supplied expiration date, followed by product testing at 3, 6, 9, 12, 15, 18, 24, and 30 months after which supplies were exhausted. Twenty specimens were fabricated for each test ( *n* = 20) with the chosen sample size designed to provide more accurate mean values and lower standard error. Flexural strength specimens were fabricated as per ISO 404919 using standardized, 2 × 2 × 25 mm stainless steel molds (Sabri Dental Enterprises; Downers Grove, IL, USA). Molds were placed onto a polyester film on the dorsal surface of a glass slab. The mold was filled with the resin composite, a second polyester strip placed, and pressure exerted using a second glass slide to form a flat and uniform surface. The resin composite was then cured with a light-emitting diode (LED) curing unit (Bluephase G2, Ivoclar-Vivadent; Amherst, NY, USA) for 20 seconds as overlapped on both sides. The VLC output was periodically assessed (~ 1000 mw/cm2) using a hand-held radiometer (BluePhase Meter II; Ivoclar Vivadent). Specimens were further refined with flash material removed using surgical scalpel blades and stored in physiologic fluid (0.2M phosphate buffered saline) in a light-proof container at 37°C and 98 ± 1% humidity. After 24 hours, specimens were tested until failure in three-point bend using a universal testing machine (Alliance RT/5; MTS Corporation, Eden Prairie, MN, USA) at 0.5 mm/min. Flexure strength (FS) was determined using the formula FS = 3FI/2bh2, where F was the maximum load recorded in Newtons, I represents the millimeter distance between supports, while b and h describe millimeter specimen width and height, respectively. Young’s modulus (flexural) was defined by linear slope of the stress/strain curve. Mean data were found to contain both an abnormal distribution and variance regularity using Shapiro–Wilk and Bartlett’s testing, respectively. Mean data were then compared using Kruskal–Wallis/Dunn’s with additional correlation analysis (Pearson’s) to identify possible similar trends between materials within each mechanical property over the study duration. All statistical analysis was performed at a 95% level of confidence (α = 0.05) using GraphPad Prism 8 (GraphPad Software; San Diego, CA, USA).

**Table 1:** Materials evaluated

Product	Classification	Constituents	Lot number
Beautifil II Shofu Dental Corporation, San Marcos, CA USA	Giomer	Bis-GMA: ~ 70% Triethylenglycol dimethacrylate: < 5% Aluminofluoro-borosilicate glass: 70% Al _2_ O _3_^a^ DL-Camphorquinone ^a^	021250
Esthet X HD Dentsply–Sirona/ Dentsply Caulk Milford, DE, USA	Nano hybrid	Hydrophobic amorphous fumed silica: < 5% Silica (amorphous): < 5% Fluoroaluminoborosilicate glass: < 50% Urethane modified Bis-GMA dimethacrylate: < 10% Polymerizable dimethacrylate resins: < 20%	111007
Filtek Supreme Ultra 3M Oral Care, St. Paul, MN, USA	Nanofill	Silane treated ceramic: 60–80% Silane treated silica: 1–10% Diurethane dimethacrylate (UDMA): 1–10% Bisphenol A polyethylene glycol diether dimethacrylate: 1–10% BISGMA: 1–10% Silane treated zirconia: 1–5% Polyethylene glycol dimethacrylate: < 5% Triethylene glycol dimethacrylate < 1%	N357235
TPH3 Dentsply-Sirona	Nano hybrid	Bariumaluminofluorosilicate glass: 49.7% Fluoroaluminoborosilicate glass: 24.6% Hydrophobic amorphous fumed and amorphous Silica: < 5% Urethane modified Bis-GMA dimethacrylate resin: 2.5 < 10% Ethoxylated bisphenol A dimethacrylate 2.5 < 10% 2,2'-Ethylendioxydiethyldimethacrylat 2.5 < 10%	1110031
Z250 3M Oral Care	Hybrid	Silane treated ceramic: 75–85% Bisphenol A polyethylene glycol diether dimethacrylate (BISEMA6): 1–10% Diurethane dimethacrylate (UDMA): 1–10% BISGMA: 1–6% Triethylenglycol dimethacrylate (TEGDMA): < 3% Aluminum oxide: 1%	N34012
Abbreviations: Bis-GMA = Bisphenol A diglycidyl ether dimethacrylate. ^a^ = content % not provided; content obtained from manufacturer literature.			

## Results

Results are displayed in Table 2 . All materials demonstrated similar flexure strength as compared with baseline for up to 15 months, with TPH3 and Z250 mean flexure values remaining similar for the entire 30-month period. Both Beautifil II and Filtek Supreme Ultra were noted to have significantly lower mean flexure strength at 18 months, with Esthet X having significantly lower values at 30 months. At 12 months, Z250 displayed a nonsignificant flexure strength increase at 12 months which decreased afterward, while both Beautifil II and Esthet X demonstrated the same trend at 15 months. Nevertheless, although some variation within the study timeframe was noted, Beautifil II, Esthet X, and TPH3 did not display significantly lower modulus values until 30 months. Z250 and Filtek Supreme Ultra both demonstrated significantly lower modulus values at 15 and 18 months, respectively. The correlation analysis results can be seen in Tables 3 4 . The flexure strength behavior between TPH3 and Esthet X HD was identified as having a strong correlation ( *p* = 0.008, r ^2^ = 0.71) with Beautifil II flexure strength behavior also identified as having a strong correlation with both TPH3 and Esthet X HD ( *p* = 0.004, r ^2^ = 0.71). Furthermore, a significant and strong correlation was found between Filtek Supreme Ultra and Esthet X HD ( *p* = 0.008, r ^2^ = 0.64) but not between Filtek Supreme Ultra and TPH3. Interestingly, correlation was not identified between Filtek Supreme Ultra and Z250 ( *p* = 0.09, r ^2^ = 0.33), both of which are produced by the same manufacturer. Filtek Supreme Ultra modulus behavior was found to have a significant correlation with Z250 ( *p* = 0.006, r ^2^ = 0.67) as well as TPH3 ( *p* = 0.045, r ^2^ = 0.45), but not Beautifil II ( *p* = 0.07, r ^2^ = 0.38). Similar to flexure strength, TPH3 and Esthet X HD maintained a strong correlation ( *p* = 0.028, r ^2^ = 0.65), but each were not as strongly correlated with Beautifil II (r ^2^ = 0.5 and r ^2^ = 0.42, respectively).

**Table 2:** Mean flexure strength and modulus results (MPa)

		Baseline	3M	6M	9M	12M	15M	18M	24M	30M
Beautifil II	Flexure strength	131.7 (12.6) A	131.6 (10.0) A	128.6 (12.6) A	125.9 (10.5) A	127.2 (14.1) A	131.3 (13.3) A	113.3 (22.4) B	112.3 (19.9) B	114.3 (17.4) B
	Modulus	11778 (1120) A	12608 (1042) A	11558 (574) A	10825 (862) B	11167 (956) A	12306 (618) A	11055 (746) A	11259 (1169) A	10196 (1105) B
Esthet X	Flexure strength	139.7 (15.9) A	142.4 (14.4) A	139.6 (7.8) A	134.5 (12.2) A	130.1 (19.8) A	143.0 (11.5) A	126.1 (23.4) A	129.7 (16.2) A	116.6 (16.2) B
	Modulus	8894 (949) A	9390 (877) A	8777 (607) A	9507 (417) A	9426 (590) A	9748 (694) B	8675 (464) A	8478 (534) A	7584 (481) B
Filtek Supreme Ultra	Flexure strength	149.9 (22.5) A	147.0 (12.2) A	144.6 (14.5) A	146.4 (17.5) A	137.1 (13.6) A	136.4 (20.6) A	133.3 (18.2) B	128.2 (16.9) B	121.3 (23.3) B
	Modulus	12393 (1535) A	11452 (731) A	11551 (952) A	11453 (1057) A	11629 (615) A	11729 (672) A	10702 (665) B	10559 (474) B	10145 (413) B
TPH3	Flexure strength	146.0 (21.0) A	156.5 (22.8) A	152.7 (20.7) A	149.7 (13.7) A	152.0 (23.2) A	164.5 (21.9) A	141.7 (18.8) A	136.8 (23.4) A	135.9 (22.1) A
	Modulus	9439 (1321) A	9299 (897) A	9158 (492) A	8638 (388) A	9798 (515) A	10016 (606) B	9174 (466) A	8935 (600) A	8225 (535) B
Z250	Flexure strength	147.0 (34.8) A	163.1 (19.4) A	155.7 (16.8) A	158.9 (16.4) A	170.9 (15.8) B	151.8 (21.2) A	152.2 (22.1) A	141.2 (23.0) A	134.5 (33.1) A
	Modulus	13903 (1680) A	13071 (691) A	13304 (1313) A	11623 (947) B	12565 (844) A	12235 (1313) B	11833 (931) B	11199 (900) B	11347 (718) B
*n* = 20; capital letters identify similar groups for each row compared with row baseline data (Kruskal—Wallis/Dunn’s, *p* < 0.05).										

**Table 3:** Correlation matrix of mean flexure strength results (Pearson’s)

	Beautifil II	Esthet X	Filtek Supreme Ultra	TPH3	Z250
Beautifil II	1.0	r = 0.84 *p* = 0.004 r ^2^ = 0.71	r = 0.81 *p* = 0.007 r ^2^ = 0.65	r = 0.84 *p* = 0.004 r ^2^ = 0.71	r = 0.58 *p* = 0.099 r ^2^ = 0.33
Esthet X	r = 0.84 *p* = 0.004 r ^2^ = 0.71	1.0	r = 0.80 *p* = 0.008 r ^2^ = 0.64	r = 0.84 *p* = 0.008 r ^2^ = 0.71	r = 0.47 *p* = 0.198 r ^2^ = 0.22
Filtek Supreme Ultra	r = 0.81 *p* = 0.007 r ^2^ = 0.65	r = 0.84 *p* = 0.008 r ^2^ = 0.71	1.0	r = 0.56 *p* = 0.109 r ^2^ = 0.31	r = 0.58 *p* = 0.095 r ^2^ = 0.33
TPH3	r = 0.84 *p* = 0.004 r ^2^ = 0.71	r = 0.84 *p* = 0.008 r ^2^ = 0.71	r = 0.56 *p* = 0.109 r ^2^ = 0.31	1.0	r = 0.64 *p* = 0.06 r ^2^ = 0.41
Z250	r = 0.58 *p* = 0.099 r ^2^ = 0.33	r = 0.47 *p* = 0.198 r ^2^ = 0.22	r = 0.58 *p* = 0.095 r ^2^ = 0.33	r = 0.64 *p* = 0.06 r ^2^ = 0.41	1.0
*n* = 20; r = Pearson’s correlation coefficient; r ^2^ = determination coefficient Correlation matrix represents comparison of all materials’ results with each other using all data points (baseline through 30 months).					

**Table 4:** Correlation matrix of mean flexural modulus results (Pearson’s)

	Beautifil II	Esthet X	Filtek Supreme Ultra	TPH3	Z250
Beautifil II	1.0	r = 0.65 *p* = 0.057 r ^2^ = 0.42	r = 0.62 *p* = 0.073 r ^2^ = 0.38	r = 0.71 *p* = 0.030 r ^2^ = 0.50	r = 0.61 *p* = 0.078 r ^2^ = 0.37
Esthet X	r = 0.65 *p* = 0.057 r ^2^ = 0.42	1.0	r = 0.71 *p* = 0.031 r ^2^ = 0.50	r = 0.72 *p* = 0.028 r ^2^ = 0.65	r = 0.34 *p* = 0.36 r ^2^ = 0.11
Filtek Supreme Ultra	r = 0.62 *p* = 0.073 r ^2^ = 0.38	r = 0.71 *p* = 0.031 r ^2^ = 0.50	1.0	r = 0.65 *p* = 0.045 r ^2^ = 0.42	r = 0.52 *p* = 0.006 r ^2^ = 0.67
TPH3	r = 0.71 *p* = 0.030 r ^2^ = 0.50	r = 0.72 *p* = 0.028 r ^2^ = 0.65	r = 0.67 *p* = 0.045 r ^2^ = 0.45	1.0	r = 0.52 *p* = 0.15 r ^2^ = 0.27
Z250	r = 0.61 *p* = 0.078 r ^2^ = 0.33	r = 0.34 *p* = 0.36 r ^2^ = 0.22	r = 0.82 *p* = 0.006 r ^2^ = 0.67	r = 0.52 *p* = 0.15 r ^2^ = 0.27	1.0
*n* = 20; r = Pearson’s correlation coefficient; r ^2^ = determination coefficient Correlation matrix represents comparison of all materials’ results with each other using all data points (baseline through 30 months).					

## Discussion

Dental direct restorative composite resins are polymers possessing both clinical and laboratory performance largely related to the configuration of the polymer’s structure and time-related degradation. 3 15 Polymer stability and its association with shelf life involve intertwining processes (e.g., chemical aging and physical aging) that are difficult to fully understand. International standards distinguish between chemical and physical degradation/aging processes, although, in practice, the processes occur simultaneously. The German Institute for Standards (DIN) 50035 identifies chemical aging as irreversible changes with molecular weight, physical structure, and/or chemical composition. 16 DIN 50035 further defines physical aging as processes that involve structural changes, organization of the molecular state, and/or changes in quotients of multicomponent systems on measurable mechanical performance that does not lead to chemical degradation. 16 However, the interactions between chemical and physical aging processes are very complex, as components of both processes intertwine and occur simultaneously with the produced degradation products also affecting the total results. 16 To wit, it can be easily envisioned where resin composite shelf life, determined by polymerization function, can be affected by both processes–illustrated by tertiary amine breakdowns (chemical aging) as well as inhibitor loss, resulting in microenvironment polymer polymerization and crystallization (physical aging). 15 Furthermore, degradation may also not be dependent on individual constituent concentration, as silane coupling agents are not a major percentage of resin composites, but silane coupling agent interaction at filler/matrix interfaces greatly influences the material performance and long-term stability. 13 14 Shelf-life determinations are based on ambient real-time testing or accelerated aging protocols, which both contain advantages and disadvantages. This evaluation followed an ambient condition and real-time protocol, as all materials were stored together in a controlled laboratory setting. This evaluation used five resin composite restorative materials that were in excess from previous research evaluations and were close to the manufacture recommended expiration date. Beautifill II is classified as a Giomer with aluminofluoro–borosilicate glass used as a filler material. Both Esthet X HD and TPH3 are classified by the manufacturer as nanohybrids, with the filler content of TPH3 and Esthet X HD being said to contain fillers consisting of barium boron fluoroalumino silicate glass, amorphous silica, and hydrophobic amorphous-fumed silica. The one nanofilled material, Filtek Supreme Ultra, is described to contain fillers listed as silane-treated ceramic and silica as well as silane treated zirconia. Z250 is classified as a hybrid consisting of silane-treated ceramic fillers. A generalized decreasing trend with all materials can be observed. Filtek Supreme Ultra is observed to follow a general decreasing flexure strength behavior, while at 12 months Z250 demonstrates a momentary flexure strength increase at 12 months. The flexure strength behavior existed at 15 months with TPH3, Esthet X HD, and to a lesser extent, Beautifil II. The flexure strength behavior between some of the materials were suggested to correlate. TPH3 and Esthet X HD were identified to have a strong correlation (r ^2^ = 0.71), with this relationship attributed to a function of similar polymer and filler components. However, Beautifil II flexure strength behavior is also identified as having a strong correlation (r ^2^ = 0.71) with both TPH3 and Esthet X HD but the resin component differs. Moreover, strong correlation was found between the flexure strength performance between Filtek Supreme Ultra and Esthet X HD (r ^2^ =0.71) but not between Filtek Supreme Ultra and TPH3 (r ^2^ = 0.31). Interestingly, correlation was not identified between Filtek Supreme Ultra and Z250 (r ^2^ = 0.33), both of which are produced by the same manufacturer. While both Z250 and Filtek Supreme Ultra essentially contain the same resin backbone and filler components, this could possibly identify a function of the mean filler size difference between the nanofilled and hybrid particles. Although material specific, the flexural modulus performance largely mirrored the behavior observed with flexure strength. Significant modulus changes were noted, but the low covariance might helped identify significant difference within each group that could unlikely be of clinical significance. Unlike flexure strength behavior, Filtek Supreme Ultra modulus behavior was found to have a significant correlation with Z250 (r ^2^ = 0.67), while TPH3 and Esthet X HD (r ^2^ = 0.65) maintained a strong correlation. The overall behavior between different material correlations when considering both flexural strength and modulus is indeed puzzling. While some reports suggest a weak correlation between flexure strength and modulus performance, 17 18 the behavior noted with the correlations noted between flexure strength and modulus may merit further investigation. Comparison of this current study with that reported in the literature is difficult due to the lack of comparative studies. Hondrum and Fernandez 4 did report shelf life flexure strength results, but it only involved two restorative resins that were not comparable with currently marketed materials. D’Alpino et al 10 evaluated Filtek Z250 and Filtek Z350XT with an accelerated aging protocol comparable to 9 months ambient storage and reported similar Z250 results of loss of flexural strength and modulus.

**Fig. 1:**
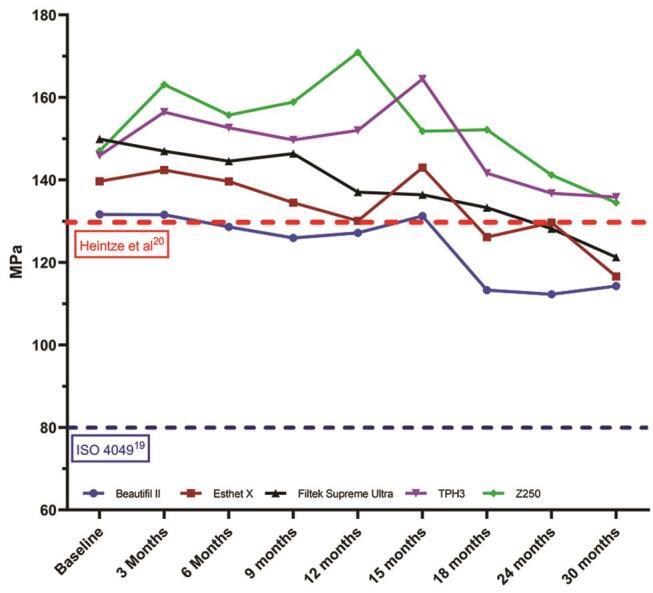
Mean flexure strength results compared with suggested minimum performance standards. *n* = 20; red dotted line annotates lower limit of 130 MPa, suggested to prevent surface wear and degradation, as suggested by Heintze et al. 20 Blue dotted line annotates minimal flexure strength required for clinical function, as agreed upon in ISO 4049 19

The null hypothesis was rejected, as changes in both flexure strength and modulus were noted over the course of the evaluation. However, an important overall consideration with evaluating data of this nature is identifying when a selected mechanical property degradation indicates that the material is no longer suitable for clinical use. For flexural strength, ISO 4049 recommends a minimal flexure strength of 80 MPa for clinical function, 19 which all materials in this study surpassed. However, recent work of Heintze et al 20 reported the results of a systematic review of 74 clinical experiments from 45 studies involving 31 different materials. While this work did not identify a correlation between flexure strength and clinical material fracture, it did suggest a strong correlation between significant resin composite surface wear and a minimal flexure strength of 130 MPa. Fig. 1 demonstrates the flexure strength results compared with suggested limits of ISO 4049 19 and that suggested by Heintze et al. 20 While all materials in this study surpassed the agreed minimal ISO 4049 flexure strength functional requirements, Beautifil II might be expected to demonstrate significant surface wear beyond 3 months after the expiration date, and Esthet X HD and Filtek Supreme Ultra might demonstrate the same after 15 and 18 months, respectively. While it would be tempting to relate the beginning of wear due to silane degradation, 13 14 21 resin composite surface wear is a multifactorial phenomenon and is usually material dependent and cannot be predicted from resin composite category, filler loading, and resin matrix. 22 23 Other effects of degradation may include reduction of fracture toughness, 24 flexure strength, 25 26 surface roughness, 27 28 all of which may cause early restoration failure.

Limitations of this study include that the materials were not thermally stressed before testing, as Rutterman et al 29 reported that flexural properties were affected by thermocycling. A further limitation was the materials evaluated were not stored in strictly controlled conditions but rather in a general laboratory environment that may represent daily temperature and humidity fluctuations as that of a clinical situation. Furthermore, baseline data was obtained approximately 1 month prior to stated expiration date, which assumes that material stability demonstrated at that time would be comparable to that obtained at material formulation. Importantly, this study identifies the complex interrelationships between resin composite constituent components and the difficulty involved with the determination of resin composite instability due to selected mechanical property evaluations. Accordingly, no guidance exists from international dental standards that detail testing methodologies for resin composite shelf-life determination. Furthermore, it is not common knowledge which manufacturer protocols are used for material shelf-life determination, and transparency with specific testing methodologies would be welcomed.

## Conclusion

Under the conditions of this study, five resin composite direct restorative materials were found to maintain flexure strength and modulus in most cases up to 15 months after the manufacturer’s recommended expiration date. However, clinicians are still advised to follow manufacturers’ recommended expiration dates, as resin composite degradation mechanisms are complex, and constituents may degrade that seriously affect restoration longevity but are not overtly identified by clinical handling characteristics and selected mechanical property testing. As no dental standards for shelf-life determination exist, manufacturers are requested provide transparency with protocols used in assigning shelf-life expiration dates.
